# FRAILSURVEY—an mHealth App for Self-Assessment of Frailty Based on the Portuguese Version of the Groningen Frailty Indicator: Validation and Reliability Study

**DOI:** 10.2196/51975

**Published:** 2025-03-07

**Authors:** Luis Midao, Mafalda Duarte, Rute Sampaio, Marta Almada, Cláudia Camila Dias, Constança Paúl, Elísio Costa

**Affiliations:** 1 RISE-Health, Biochemistry Lab, Faculty of Pharmacy, University of Porto Porto Portugal; 2 Porto4Ageing—Competences Centre on Active and Healthy Ageing, Faculty of Pharmacy, University of Porto Porto Portugal; 3 ICBAS—School of Medicine and Biomedical Sciences, University of Porto Porto Portugal; 4 RISE-Health, Department of Behavioural Sciences, School of Medicine and Biomedical Sciences, University of Porto Porto Portugal; 5 ISAVE—Superior Institute of Health, Amares Braga Portugal; 6 RISE-Health, Department of Biomedicine, Faculty of Medicine of the University of Porto Porto Portugal; 7 Knowledge Management Unit, Faculty of Medicine of the University of Porto (FMUP) Porto Portugal; 8 CINTESIS@RISE, Department of Community Medicine, Information and Health Decision Sciences (MEDCIDS), Faculty of Medicine of the University of Porto (FMUP) Porto Portugal

**Keywords:** frailty, mHealth, assessment, validation, GFI, reliability, self-assessment, Groningen Frailty Indicator, FRAILSURVEY, mobile phone

## Abstract

**Background:**

Portugal is facing the challenge of population ageing, with a notable increase in the proportion of older individuals. This has positioned the country among those in Europe with a high prevalence of frailty. Frailty, a geriatric syndrome characterized by diminished physiological reserve and heightened vulnerability to stressors, imposes a substantial burden on public health.

**Objective:**

This study seeks to address two primary objectives: (1) translation and psychometric evaluation of the European Portuguese version of the Groningen Frailty Indicator (GFI); and (2) development and evaluation of the FRAILSURVEY app, a novel assessment tool for frailty based on the GFI. By achieving these objectives, the study aims to enhance the accuracy and reliability of frailty assessment in the Portuguese context, ultimately contributing to improved health care outcomes for older individuals in the region.

**Methods:**

To accomplish the objectives of the study, a comprehensive research methodology was used. The study comprised 2 major phases: the initial translation and validation of the GFI into European Portuguese and the development of the FRAILSURVEY app. Following this, an extensive examination of the app’s validity and reliability was conducted compared with the conventional paper version of the GFI. A randomized repeated crossover design was used to ensure rigorous evaluation of both assessment methods, using both the paper form of the GFI and the smartphone-based app FRAILSURVEY.

**Results:**

The findings of the study revealed promising outcomes in line with the research objectives. The meticulous translation process yielded a final version of the GFI with robust psychometric properties, ensuring clarity and comprehensibility for participants. The study included 522 participants, predominantly women (367/522, 70.3%), with a mean age of 73.7 (SD 6.7) years. Psychometric evaluation of the European Portuguese GFI in paper form demonstrates good reliability (internal consistency: Cronbach a value of 0.759; temporal stability: intraclass correlation coefficient=0.974) and construct validity (revealing a 4D structure explaining 56% of variance). Evaluation of the app-based European Portuguese GFI indicates good reliability (interinstrument reliability: Cohen k=0.790; temporal stability: intraclass correlation coefficient=0.800) and concurrent validity (*r*=0.694; *P*<.001).

**Conclusions:**

Both the smartphone-based app and the paper version of the GFI were feasible and acceptable for use. The findings supported that FRAILSURVEY exhibited comparable validity and reliability to its paper counterpart. FRAILSURVEY uses a standardized and validated assessment tool, offering objective and consistent measurements while eliminating subjective biases, enhancing accuracy, and ensuring reliability. This app holds promising potential for aiding health care professionals in identifying frailty in older individuals, enabling early intervention, and improving the management of adverse health outcomes associated with this syndrome. Its integration with electronic health records and other data may lead to personalized interventions, improving frailty management and health outcomes for at-risk individuals.

## Introduction

### Background

Portugal is currently confronting a significant population ageing phenomenon, placing it among the European countries with one of the highest percentages of older individuals. As of 2021, the ageing index reached 182, meaning there were 182 people aged 65 years or older for every 100 individuals aged 0-14 years. This ageing trend results from various contributing factors, such as low birth rates, extended life expectancy, and emigration of young people. According to the 2011 census, 19% of the Portuguese population were aged 65 years or older. A substantial increase was denoted to 23.4% (2,423,639/10,343,066) in the 2021 census. Projections indicate that this percentage will continue to rise in the upcoming years. Estimations forecast that by 2080, the proportion of people aged 65 years and older will escalate to 37.5% of the total population [1-4]. This demographic change has significant implications for the country’s economy, social security, and health care systems. The increasing number of older people will require greater resources to provide for their care and support, including health care services, long-term care facilities, and home care services. At the same time, the working-age population will be under pressure to support the social security system as the number of retirees increases and the number of workers decreases. As the proportion of older people grows, so does the prevalence of age-related diseases, comorbidities, and geriatric syndromes. Portugal ranks among the European countries with the highest prevalence of frailty (20.7%) [5,6].

Frailty is characterized by a reduced physiologic reserve and increased susceptibility to stressors, leading to an elevated risk of adverse health outcomes such as falls, hospitalization, disability, and death. Typically associated with ageing, frailty may encompass a blend of physical, cognitive, and psychosocial factors, making it a complex and multifaceted condition, and, therefore, identifying and addressing frailty is essential for preventing comorbidities, optimizing health outcomes, and improving the quality of life in older adults [7-11]. However, evaluating frailty poses challenges due to its multifactorial nature, resulting in various tools and methods commonly used for its assessment. It is crucial to recognize that every assessment tool is flawed, lacks standardization, and different approaches may be more suitable for specific populations or settings [10,12].

The Groningen Frailty Indicator (GFI) stands out for its comprehensive assessment for evaluating frailty in older adults. With its rapid administration and validated reliability in diverse populations, the GFI serves as a predictive tool for adverse outcomes such as hospitalization and mortality. Its multidimensional approach offers a nuanced understanding of frailty, making it invaluable for routine screening in clinical practice, empowering health care professionals to craft suitable care plans for their patients [13-16]. Covering physical, psychological, social, and cognitive aspects, the paper version of the GFI has undergone validation in diverse settings, such as among Romanian community-dwelling older adults [17] and Chinese nursing home residents [18], and different populations, such as patients with rheumatoid arthritis [19], patients with end-stage hip and knee osteoarthritis [20], and head and neck cancer surgery patients [21], confirming its accuracy, consistency, and predictive value. Distinguishing itself from other instruments, the GFI boasts a straightforward administration process, requiring no more than 10 minutes to complete. Furthermore, as a noninvasive instrument, individuals can complete it through self-reporting or seek guidance from a trained health professional, if necessary [15,16]. There are already some versions in European Portuguese of frailty scales, such as the Edmonton Frailty Scale [22], Program on Research for Integrating Services for the Maintenance of Autonomy questionnaire (PRISMA-7) [23], FRAIL Scale [24], Clinical Frailty Scale [25], SUNFRAIL [26], and Tilburg Frailty Indicator [27]; however, some are not yet validated for the Portuguese population. To our knowledge, there is still no study on the translation and validation of the GFI into European Portuguese.

Despite being commonly used, paper-based questionnaires have several disadvantages: manual data entry is time-consuming and leads to potential errors; accessibility is limited as they must be physically distributed and collected; data security may be compromised; and incomplete or illegible responses and reduced data analysis capabilities are common challenges. Furthermore, they lack interactive features that enhance engagement and data quality, such as in digital formats [28]. Smartphone-based assessments aim to develop a user-friendly tool that streamlines screening and monitoring by scoring data [29,30]. Such an approach has the potential to increase health literacy resulting in cost savings. Nevertheless, it is crucial to validate the smartphone-based apps to ensure their accuracy and reliability compared with the original paper questionnaires, as there may be possible response bias between the 2 versions [31].

### Aim

Given the high burden of frailty in Portugal and to contribute to improving frailty assessment, enhancing health care delivery, and addressing the specific needs of older adults in Portugal, this study aimed to translate and validate the GFI into European Portuguese. Then, this study also aimed to examine the validity and reliability of the digital version of the GFI, the app FRAILSURVEY.

## Methods

This is a quantitative, exploratory research that involves analysis of psychometric data and that was developed in 2 major phases: initially, the GFI was translated and validated into European Portuguese, and the application FRAILSURVEY was developed and its validity and reliability studied as a mobile health (mHealth) tool for frailty assessment.

### Translation and Validation of the GFI

The GFI is a short, easy-to-use instrument that has proven to be a good alternative for assessing frailty in the population aged 65 years or older [32]. Textbox 1 indicates the domains assessed in the GFI scale, as well as scoring items. The self-assessment version of the GFI published in 2013 was used, composed of 15 items and divided into 4 domains [33]. The physical domain has 9 questions related to toileting, shopping, mobility functions, dressing, physical fitness, vision, hearing, unexplained weight loss, and medication use. The cognitive domain includes 1 item related to cognitive concerns. The social domain consists of 3 questions about emotional isolation, social relations, and social support. The psychological domain comprises 2 questions related to mood. Each item has a score of 0 or 1 attributed, with 1 indicating a dependency problem. For example, if the participant cannot dress or undress without help, he would score 1. The total score ranged from 0 to 15, and higher scores indicate greater levels of frailty. The originally proposed cutoff point of 4 or higher represents frailty [16].

**Self-assessment version of the Groningen Frailty Indicator (GFI): with 15 items divided by 4 main domains (physical, cognitive, social, and psychological), the GFI scores between 0 and 15. People with scores of 4 and above are considered frail [33].**

**Physical domain**

Are you able to carry out these tasks single-handedly and without any help? (The use of help resources such as a walking stick, walking frame, or wheelchair is considered independent.)

1. Shopping

2. Walking around outside (around the house or to the neighbors)

3. Dressing and undressing

4. Going to the toilet

5. What mark do you give yourself for physical fitness? (Scales 0-10)

6. Do you experience problems in daily life due to poor vision?

7. Do you experience problems in daily life due to being hard of hearing?

8. During the last 6 months have you lost a lot of weight unwillingly? (3 kg in 1 month or 6 kg in 2 months)

9. Do you take 4 or more different types of medicine?

**Cognitive domain**

10. Do you have any complaints about your memory?

**Social domain**

11. Do you sometimes experience emptiness around yourself?

12. Do you sometimes miss people around yourself?

13. Do you sometimes feel abandoned?

**Psychological domain**

14. Have you recently felt downhearted or sad?

15. Have you recently felt nervous or anxious?

**Scoring**

Questions 1-4: Yes=0; No=1

Question 5: 0-6=1; 7-10=0

Questions 6-15: No=0; Yes=1

For the development of the European Portuguese version of the GFI, the self-report instrument guidelines defined by the ISPOR Task Force for Translation and Cultural Adaptation were followed [34]. Briefly, the translation was done by 2 technicians, with experience in this type of task, and the final version of each item (of the 15) always resulted from complete agreement between them. After that an English technician carried out the back-translation, and a consensus was reached as the back-translation version was in line with the original version.

### Developing the Smartphone-Based App of the GFI

The smartphone-based app was developed for both Android (the operating system created by Google) and iOS (created by Apple) systems. FRAILSURVEY is a questionnaire that comprises 2 sets of questions: 19 questions about sociodemographic data; social resources; and self-perception of health, nutrition, medication, psychosocial and cognitive status, and time occupation, plus a set of 15 questions used to assess frailty status, the European Portuguese version of the GFI. FRAILSURVEY also uses the originally proposed cutoff point of 4 or higher representing frailty [16].

FRAILSURVEY development was based on an accessibility principle for older adults to guarantee greater autonomy to use it. Therefore, considerations such as vision, hearing, motor control, and cognition were considered. Considering the vision of the older population, the app features a typographic font designed for low-vision people, APHont, developed by the American Printing House for the Blind. It also presents a way to increase or decrease the font size, as well as the possibility of listening to the audio information.

**Figure 1:**
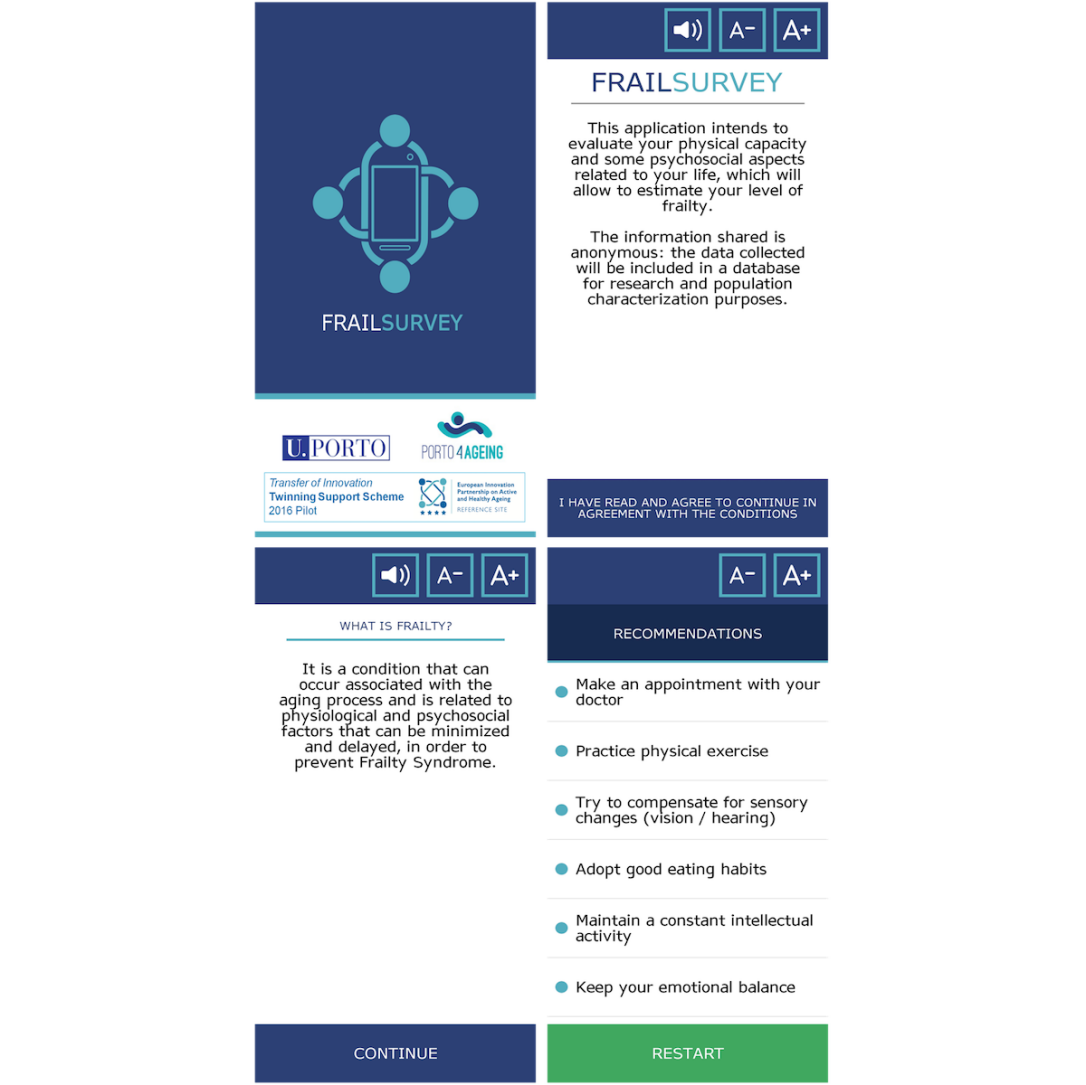
Printscreens of the FRAILSURVEY app.

The results obtained classify the person as robust or frail. The app displays tailored recommendations if the person is classified as frail. The recommendations revolve around aspects such as physical exercise, a healthy diet, and social networks, among others (Figure 1). The app generates an anonymous database for research purposes.

### Study Design

A randomized repeated crossover design was used with the paper form of the GFI and the smartphone-based app FRAILSURVEY, as used in other similar studies [35]. This design entailed the dual administration of both assessment methods to each participant on distinct occasions, separated by a 1-week interval. The order of presentation for the assessments was randomized to mitigate any potential sequence effects. In evaluating test-retest reliability, a subset of participants was randomly chosen from the larger cohort to participate in retest assessments approximately 2 weeks after their initial interaction. These retest assessments encompassed both the paper-based form and the application [36].

### Ethical Considerations

Ethical clearance for the research was granted by the ethical review board of the faculty of pharmacy, University of Porto (33-11-2017). Before their involvement, the rights and responsibilities of participants were comprehensively elucidated, and written consent was obtained from all individuals. Participation was voluntary and no compensation was provided. Throughout the process of data collection and entry, strict confidentiality measures were upheld and all data were anonymized.

### Participants

Between February 2019 and February 2020, older adults living in the district of Porto, Portugal, were invited in cultural and sports associations, day centers, and nursing homes to take part in this study. A total of 522 participants were recruited for this study, using a convenience sampling method. The sample included a diverse population of community-dwelling older individuals, residents of nursing homes, and older adults living in their own homes but availing daily care facilities. The inclusion criteria encompassed individuals aged 65 years and older, regardless of gender, who could independently complete the assessments. The sample exceeded the sample size required to be 10 times larger than the number of GFI items [37]. For test-retest reliability, 10% (52/522) of the participants were recruited randomly, but only 24 participants accepted to perform the retests and, therefore, were included in the test-retest analysis [36].

### Statistical Analysis

Categorical variables were summarized as absolute and relative frequencies. Descriptive statistics were used to report the subject characteristics of the study sample. Differences between different subgroups were studied, that is, gender, age, marital status, education, and financial status. GFI scores (from 0 to 15) were presented as mean (SD), and differences between subgroups were tested through the Kruskal-Wallis test. GFI classification (nonfrail and frail) was presented as frequencies, and differences between different subgroups were tested through the Mann-Whitney *U* test. To address the issue of multiple comparisons, adjustments were made, and the Bonferroni correction was applied to all statistical tests involving associations across multiple demographic variables.

The psychometric properties of the European Portuguese version of the GFI were assessed as follows: internal consistency referred to the extent to which the items within a scale or questionnaire consistently measure the same construct and was assessed by Cronbach a. The retest was conducted 2 weeks after the initial contact with the participant for assessing temporal stability. A 2-week interval allows for a sufficient gap between the 2 administrations to minimize potential memory effects and recall bias while maintaining a relatively short time frame. The most commonly used statistical measure to evaluate temporal stability is the intraclass correlation coefficient (ICC), and therefore it was used in this study. The ICC value ranges between 0 and 1, with higher values indicating greater temporal stability. Values above 0.9 suggest excellent temporal stability, while values between 0.75 and 0.9 indicate good temporal stability and values between 0.5 and 0.75 show moderate temporal stability [38]. Construct validity was assessed by performing exploratory factor analysis with principal components and varimax rotation methods [39].

The psychometric properties of the FRAILSURVEY were assessed as follows: interinstrument reliability refers to the consistency of GFI scores obtained by the same person between the paper and smartphone versions. Cohen k was used to assess it. Cohen suggested the k result be interpreted as follows: values ≤0 as indicating no agreement, 0.01-0.20 as none to slight (light), 0.21-0.40 as fair (fair), 0.41-0.60 as moderate (moderate), 0.61-0.80 as substantial (considerable), and 0.81-1.00 as almost perfect agreement [40]; temporal stability was evaluated by the ICC, as described previously; and the concurrent validity was studied through Pearson correlation coefficient that was used to quantify the relationship between the scores obtained in the app and in paper and with the app, the scores from the established criterion measure, and the scores from the instrument we intended to validate. Pearson correlation coefficient assesses the strength and direction of the correlation, indicating the degree to which the 2 measures agree or disagree. The validity assessment in this study was based on demonstrating the absence of significant differences in the total score of the GFI and each item of the GFI. Two-tailed *t* tests were used, and a *P* value of <.05 was considered statistically significant. The significance level used was .05. Data were analyzed using SPSS Statistics (version 28.0; IBM Corp).

## Results

### European Portuguese Version of the GFI

The English version of the GFI tool was translated into European Portuguese by 2 native Portuguese speakers. The back-translation was done by a professional service provider who knew nothing about the purpose of the research. A panel of experts assessed the similarity of the versions and reviewed and resolved all discrepancies and ambiguities by consensus. To assess the comprehensibility of the language and wording, the revised preliminary European Portuguese version was used with a pilot sample of 10 native Portuguese-speaking older adults. The final European Portuguese version of the GFI was defined and used in the validation process. No changes were made to the version used, as the items were judged to be clear and easy to understand. In the end, the final version was produced (Table S1 in Multimedia Appendix 1).

### Participants

In this study, 522 participants were included, with a mean average age of 73.7 (SD 6.7) years and consisted mainly of women (367/522, 70.3%). Almost two-thirds of the sample were aged between 65 and 74 years, most participants were married (311/522, 59.6%), the majority had only primary school completed (283/522, 54.2%), and 59.2% (309/522) reported having moderate money to meet their needs (Table 1). Using the paper version of the European Portuguese version of the GFI, the average score was 4.57, significantly higher in women (5.05) than in men (3.43; *P*<.001). The score increases significantly with age, with higher scores being more present in older people (GFI=3.73 for participants aged between 65 and 74 years; GFI=5.58 for participants aged between 75 and 84 years, and; GFI=7.77 for participants aged 85 years or older; *P*<.001). There are also significant differences in the mean GFI scores according to marital status, with married (3.67) and divorced people (4.62) having lower values while widowed (6.02) or single people (6.13) having higher values (*P*<.001). Frailty is also related to education. We reported that higher frailty scores are associated with lower levels of education, and with the economic level, with greater scores of frailty in people with lower economic levels.

**Table 1:** Participants’ sociodemographic characteristics according to their frailty status: 522 participants were included in this randomized repeated crossover design study.

			Variables, n (%)		Portuguese GFI^a^, mean (SD)		*P* value (Kruskal-Wallis test)		Nonfrailty, n (%)		Frailty, n (%)		*P* value (Mann-Whitney *U* test)	
**Sex**							<.001							<.001
	Female	367 (70.3)		5.05 (3.04)				127 (34.6)		240 (65.4)				
	Male	155 (29.7)		3.43 (2.702)				89 (57.4)		66 (42.6)				
**Age (years)**							<.001							<.001
	65-74	341 (65.3)		3.73 (2.68)				181 (53.1)		160 (46.9)				
	75-84	134 (25.7)		5.58 (2.91)				31 (23.1)		103 (76.9)				
	85+	47 (9.0)		7.77 (2.86)				4 (8.5)		43 (91.5)				
**Marital status**							<.001							.003
	Single	32 (6.1)		6.13 (3.24)				6 (18.8)		26 (81.3)				
	Married	311 (59.6)		3.67 (2.47)				161 (51.8)		150 (48.2)				
	Widowed	158 (30.3)		6.02 (3.34)				38 (24.1)		120 (75.9)				
	Divorced	21 (4.0)		4.62 (2.94)				11 (52.4)		10 (47.6)				
**Education**							<.001							<.001
	Illiterate	36 (6.9)		7.03 (3.11)				5 (13.9)		31 (86.1)				
	Unfinished primary school	80 (15.3)		5.90 (2.95)				19 (23.8)		61 (76.3)				
	Primary school	283 (54.2)		4.29 (2.97)				128 (45.2)		155 (54.8)				
	High school	92 (17.6)		3.52 (2.32)				47 (51.1)		45 (48.9)				
	Higher education	31 (5.9)		4.00 (3.24)				17 (54.8)		14 (45.2)				
**Do you think you have enough money to meet your needs?**							<.001							<.001
	Nothing	12 (2.3)		5.00 (3.77)				6 (50.0)		6 (50.0)				
	Little	190 (36.4)		5.89 (3.20)				47 (24.7)		143 (75.3)				
	Moderately	309 (59.2)		3.78 (2.63)				158 (51.1)		151 (48.9)				
	Quite enough	11 (2.1)		(1.96)				4 (45.5)		6 (54.5)				

^a^GFI: Groningen Frailty Indicator.

According to the GFI frailty classification, using the cutoff point of 4 [16], 58.6% of the people included in this study are frail. Frailty is significantly greater in women (65.4% vs 42.6% in men; *P*<.001), in older people (91.5% in people aged 85 years and older vs 76.9% in people aged 75-84 years vs 46.9% in people aged 65 and 74 years; *P*<.001), and in single people and widows (81.3% and 75.9%, respectively, vs 48.2% in married people and 47.6% in divorced people; *P*<.001). The proportion of frail people is also significantly higher for people with the lowest level of education and at lower economic levels.

### Item Descriptive Analysis and Missing Data

Of the 15 indicators, the most prevalent among all people are the items that assess nervousness and anxiety (present in 320/521, 61.4% of the participants), problems in daily life due to poor vision reported by 56% (291/520) of the participants, and polypharmacy reported by 53.6% (279/522; Table 2). The least prevalent items are mobility issues, more specifically, the inability to dress and undress alone (51/521, 9.8%) and to use the bathroom without help (41/522, 7.9%), and the feeling of abandonment (37/522, 7.1%).

**Table 2:** Groningen Frailty Indicator: missing values and criteria fulfillments: of the 522 participants, questions 3, 4, 14, and 15 had 1 missing value; questions 6 and 9 had 2 missing values; and question 5 had 10 missing values.

Question	Fulfills criteria, n (%)	Missing values, n
1. Shopping	522 (18.4)	0
2. Walking around outside (around the house or to the neighbors)	522 (13.0)	0
3. Dressing and undressing	521 (9.8)	1
4. Going to the toilet	521 (7.9)	1
5. How do you rate your physical fitness? (scale 0-10)	512 (35.5)	10
6. Do you experience problems in daily life due to poor vision?	520 (56.0)	2
7. Do you experience problems in daily life due to being hard of hearing?	522 (35.1)	0
8. During the last 6 months, did you lose a lot of weight unwillingly? (3 kg in 1 month or 6 kg in 2 months)	522 (11.5)	0
9. Do you take 4 or more different types of medicine?	520 (53.6)	2
10. Do you have any complaints about your memory?	522 (35.2)	0
11. Do you sometimes experience an emptiness around you?	522 (39.1)	0
12. Do you sometimes miss people around you?	522 (28.0)	0
13. Do you sometimes feel abandoned?	522 (7.1)	0
14. Did you feel downhearted or sad recently?	521 (46.6)	1
15. Did you feel nervous or anxious recently?	521 (61.4)	1

Of the 522 participants, there are only 18 missing values in 7 of the 15 GFI questions. The average of missing values per participant is 0.03. Only 1 participant has missing values for 2 GFI questions, while the other 17 missing values come from 17 different participants. The question that has the most missing values (1.9%) is the question that asks the participant how he rates his physical fitness (from 0 to 10). The small proportion of missing data, constituting only 0.23% of the total values, suggests that it is improbable for these missing values to significantly impact the overall patterns or conclusions of the analysis, while the large sample size (522 participants) ensures that the study maintains sufficient statistical power to detect meaningful effects or relationships despite the negligible reduction in sample size attributable to missing data.

### Psychometric Evaluation of the European Portuguese Version of the GFI (Paper)

#### Internal Consistency

The obtained Cronbach a value of 0.759 suggests a moderate level of internal consistency and no item was dropped (Table 3) [33].

#### Temporal Stability

The values varied between 0.504 and 1.000, being an overall value of 0.974 (Table 3).

**Table 3:** Psychometric Evaluation of the European Portuguese version of the GFI^a^—reliability analysis (internal consistency and temporal stability): no item was dropped and the Cronbach a value of 0.759 suggests a moderate level of internal consistency.

Question	Internal Consistency			Temporal stability, ICC^b^
	Scale average if the item is removed	Scale variation if the item is removed	Cronbach a if the item is removed	
1. Shopping	4.40	7.876	0.729	1.000
2. Walking around outside (around the house or to the neighbors)	4.46	8.189	0.737	0.947
3. Dressing and undressing	4.49	8.362	0.741	1.000
4. Going to the toilet	4.51	8.504	0.744	0.909
*5.* How do you rate your physical fitness? (scale 0-10)	4.23	7.882	0.740	0.940
6. Do you experience problems in daily life due to poor vision?	4.02	8.438	0.764	1.000
7. Do you experience problems in daily life due to being hard of hearing?	4.24	8.381	0.759	1.000
8. During the last 6 months, did you lose a lot of weight unwillingly? (3 kg in 1 month or 6 kg in 2 months)	4.47	8.952	0.765	1.000
9. Do you take 4 or more different types of medicine?	4.05	8.087	0.751	1.000
10. Do you have any complaints about your memory?	4.24	8.257	0.755	0.911
11. Do you sometimes experience an emptiness around you?	4.20	7.795	0.737	0.958
12. Do you sometimes miss people around you?	4.30	8.014	0.742	0.727
13. Do you sometimes feel abandoned?	4.51	8.683	0.751	0.880
14. Did you feel downhearted or sad recently?	4.13	7.547	0.727	1.000
15. Did you feel nervous or anxious recently?	3.97	8.092	0.749	0.504
Total			0.759	0.974

^a^GFI: Groningen Frailty Indicator.

^b^ICC: intraclass correlation coefficient.

#### Construct Validity

The suitability of the factor analysis by checking the existence of significant correlations between the items was confirmed by Kaiser-Meyer-Olkin test (0.799) and by Bartlett’s Test of Sphericity (*χ*²_105_=2074.7; *P*<.001). The Kaiser-Meyer-Olkin measure assesses the suitability of data for factor analysis by evaluating proportions of variance in variables potentially linked to underlying factors, with higher values indicating better fit, typically above 0.6 or 0.7. Its advantage lies in providing a simple index for researchers to interpret and make decisions. Bartlett’s Test of Sphericity validates the interrelationship of variables necessary for factor analysis, testing the null hypothesis of no correlation between variables, with a significant result (*P*<.05) confirming the suitability of the dataset for analysis factorial [41].

Evaluation of the scree plot and the size of the eigenvalues strongly suggest that the GFI has a 4D structure, explaining 56.0% of the variance (Table 4). This analysis produced four subscales: (1) Daily Activities and Physical Fitness (items 1-5), (2) Nourishment, (3) Health Problems (items 6, 7, 9, and 10), and (4) Psychosocial Functioning (items 11-15).

**Table 4:** Psychometric Evaluation of the European Portuguese version of the GFI^a^—validity analysis (construct validity): exploratory factor analysis structure of the Portuguese version of the GFI: loading for each factor and each item in the model with 4 factors after an orthogonal varimax rotation and factor extraction using principal components (n=502): GFI has a 4D structure, explaining 56.0% of the variance.

Items			Factors^b^						
			1		2		3		4
**Daily Activities and Physical Fitness**									
	1. Shopping	0.822		0.167		0.165		–0.009	
	2. Walking around outside (around the house or to the neighbors)	0.863		0.084		0.087		–0.038	
	3. Dressing and undressing	0.880		0.076		0.002		0.018	
	4. Going to the toilet	0.872		0.028		–0.028		0.061	
	5. How do you rate your physical fitness? (scale 0-10)	0.505		0.179		0.279		–0.034	
**Health Problems**									
	6. Do you experience problems in daily life due to poor vision?	–0.008		0.033		0.511		0.325	
	7. Do you experience problems in daily life due to being hard of hearing?	0.061		–0.073		0.705		0.094	
	9. Do you take 4 or more different types of medicine?	0.183		0.134		0.563		–0.172	
	10. Do you have any complaints about your memory?	0.062		0.206		0.465		0.073	
**Nourishment**									
	8. During the last 6 months, did you lose a lot of weight unwillingly? (3 kg in 1 month or 6 kg in 2 months)	–0.053		0.041		0.153		0.832	
**Psychosocial Functioning**									
	11. Do you sometimes experience an emptiness around you?	0.092		0.817		0.058		–0.007	
	12. Do you sometimes miss people around you?	0.029		0.778		0.042		0.152	
	13. Do you sometimes feel abandoned?	0.270		0.432		–0.092		0.368	
	14. Did you feel downhearted or sad recently?	0.258		0.676		0.190		–0.005	
	15. Did you feel nervous or anxious recently?	–0.008		0.497		0.389		–0.214	
Percentage of total variance explained for each factor			N/A^c^		13.50		8.66		6.67

^a^GFI: Groningen Frailty Indicator.

^b^Percentage of total variance explained for the 4 extracted factors=56%; Kaiser-Meyer-Olkin=0.799 and Bartlett’s Test of Sphericity <0.001.

^c^N/A: not applicable.

### Psychometric Evaluation of the FRAILSURVEY—an mHealth Tool for Frailty Assessment

#### Interinstrument Reliability

The values varied between 0.100 and 0.634, being an overall value of 0.790 (Table 5). From the 15 items, 2 items had a substantial agreement, 5 had a moderate agreement, 6 had a fair agreement, 2 had none to a slight agreement, and the overall score had an almost perfect agreement.

**Table 5:** Psychometric Evaluation of the FRAILSURVEY—reliability analysis (interinstrument reliability and temporal stability)a.

Question	Interinstrument reliability					Temporal stability, ICC^b^
	SE values	Observed k values	Lower limit (95% CI)	Upper limit (95% CI)		
1. Shopping	0.045	0.634	0.546	0.722	0.793	
2. Walking around outside (around the house or to the neighbors)	0.053	0.608	0.503	0.712	0.867	
3. Dressing and undressing	0.069	0.522	0.387	0.657	0.958	
4. Going to the toilet	0.079	0.468	0.314	0.622	0.875	
5. How do you rate your physical fitness? (scale 0-10)	0.043	0.419	0.335	0.503	0.788	
6. Do you experience problems in daily life due to poor vision?	0.042	0.266	0.182	0.349	0.958	
7. Do you experience problems in daily life due to being hard of hearing?	0.045	0.289	0.201	0.378	0.798	
8. During the last 6 months, did you lose a lot of weight unwillingly? (3 kg in 1 month or 6 kg in 2 months)	0.086	0.100	0.000	0.270	0.958	
9. Do you take 4 or more different types of medicine?	0.042	0.337	0.256	0.419	0.913	
10. Do you have any complaints about your memory?	0.045	0.236	0.147	0.324	0.724	
11. Do you sometimes experience an emptiness around you?	0.041	0.454	0.374	0.533	0.857	
12. Do you sometimes miss people around you?	0.047	0.336	0.245	0.428	0.667	
13. Do you sometimes feel abandoned?	0.099	0.188	0.000	0.381	0.793	
14. Did you feel downhearted or sad recently?	0.039	0.443	0.366	0.520	0.718	
15. Did you feel nervous or anxious recently?	0.043	0.328	0.243	0.413	0.589	
GFI^c^ score	0.027	0.790	0.737	0.844	0.800	

^a^Evaluation of the app-based European Portuguese GFI indicates good reliability (interinstrument reliability: Cohen k=0.790; temporal stability: ICC=0.800).

^b^ICC: intraclass correlation coefficient.

^c^GFI: Groningen Frailty Indicator.

#### Temporal Stability

The retest was administered 2 weeks after the initial contact with the participant to evaluate the temporal stability of the measurements. The ICC values varied between 0.589 and 0.958, being an overall value of 0.800 (Table 5).

#### Concurrent Validity

All values were positive, ranging from 0.101 to 0.634, and the total score was 0.694 (Table 6). These results demonstrate a positive correlation between responses in both formats, paper, and app.

**Table 6:** Psychometric evaluation of the FRAILSURVEY—validity analysis (concurrent validity): a positive correlation between responses in both formats, paper, and app was observed.

Question	N	FRAILSURVEY, mean (SD)	Paper, mean (SD)	*r* value	*P* value
1. Shopping	522	0.20 (0.40)	0.18 (0.39)	0.634	<.001
2. Walking around outside (around the house or to the neighbors)	522	0.15 (0.36)	0.13 (0.34)	0.609	<.001
3. Dressing and undressing	521	0.10 (0.30)	0.10 (0.30)	0.522	<.001
4. Going to the toilet	521	0.09 (0.28)	0.08 (0,27)	0.468	<.001
5. How do you rate your physical fitness? (scale 0-10)	512	0.35 (0.48)	0.36 (0.48)	0.419	<.001
6. Do you experience problems in daily life due to poor vision?	520	0.52 (0.50)	0.56 (0.50)	0.266	<.001
7. Do you experience problems in daily life due to being hard of hearing?	522	0.33 (0.47)	0.35 (0.48)	0.290	<.001
8. During the last 6 months, did you lose a lot of weight unwillingly? (3 kg in 1 month or 6 kg in 2 months)	522	0.10 (0.30)	0.11 (0.32)	0.101	.02
9. Do you take 4 or more different types of medicine?	520	0.55 (0.50)	0.54 (0.50)	0.337	<.001
10. Do you have any complaints about your memory?	522	0.38 (0.49)	0.35 (0.48)	0.236	<.001
11. Do you sometimes experience an emptiness around you?	522	0.38 (0.49)	0.39 (0.49)	0.454	<.001
12. Do you sometimes miss people around you?	522	0.32 (0.47)	0.28 (0.45)	0.338	<.001
13. Do you sometimes feel abandoned?	522	0.08 (0.28)	0.07 (0.26)	0.189	<.001
14. Did you feel downhearted or sad recently?	521	0.50 (0.50)	0.47 (0.50)	0.444	<.001
15. Did you feel nervous or anxious recently?	521	0.64 (0.48)	0.61 (0.49)	0.329	<.001
Total score	522	4.67 (3.01)	4.57 (3.04)	0.694	<.001

## Discussion

### Principal Findings

Translating and validating frailty assessment tools are vital for cross-cultural research, ensuring inclusivity and diversity, and allowing researchers and health care professionals to identify frailty in older adults independently or irrespective of linguistic and cultural backgrounds [42]. Translated versions enhance measurement accuracy and consistency by retaining the psychometric properties and measurement validity. This facilitates the development of comprehensive public health policies and targeted interventions, improving care and quality of life for ageing populations worldwide [43]. Overall, translating and validating frailty assessment tools are crucial in advancing research, clinical practice, and public health initiatives related to frailty [44]. Frailty assessment tools are valuable for identifying at-risk individuals and tailoring interventions to their needs. By identifying frailty early, health care practitioners can develop strategies to prevent functional decline and mitigate adverse health outcomes [45].

In this study, the translation of the GFI into European Portuguese underwent rigorous processes, resulting in a final version with strong psychometric properties. The translated version proved to be clear and easily understandable.

Among the 522 participants in the study, the majority were women, which aligns with typical demographic patterns in this type of research. In addition, most participants were aged 65-74 years, were either married or widowed, had completed only primary education, and had moderate money to meet their needs.

The mean GFI score was 4.57 in this study, whereas validation studies conducted in China, Romania, and Lebanon were 4.60, 5.68, and 6.80, respectively [17,18,46]. Consistent with previous research, the average score was significantly higher in women than in men [5,12,17,46]. As anticipated, there was a significant positive correlation between age and GFI score and, additionally, marital status showed significant differences, with singles and widowers having higher scores [47]. Frailty also demonstrated significant associations with lower education levels and lower economic statuses, as already shown [48].

The GFI comprises 15 frailty indicators and the most prevalent were polypharmacy, vision problems, and feelings of anxiety or nervousness, all reported by more than 50% of the participants. Indeed, the link between polypharmacy and frailty has been established through a study using SHARE data, which explored the connection between mortality, frailty, and polypharmacy across 18 countries. According to these findings, 59.4% of the identified frail population was found to be taking multiple medications, a percentage that closely aligns with the results obtained in this study, where 53.6% of the population were under polypharmacy [5]. Frailty and poor vision are interconnected conditions with a bidirectional relationship, as already described [49]. Poor vision can contribute to frailty by impairing balance, mobility, and daily functioning, increasing the risk of falls and social isolation. It has already been described that people with visual impairment have 3.7 greater odds of being frail [49]. Anxiety symptoms, reported by 61.4% (320/521) of the participants of this study, were reported to be notably more prevalent among older adults with frailty than among those who are robust, which has also been reported elsewhere [50].

The psychometric evaluation of the European Portuguese version of the GFI demonstrated good reliability and validity. In previous psychometric studies assessing the internal consistency of the GFI, Cronbach α values ranged from 0.680 to 0.810. Nevertheless, in this study, the obtained value was 0.759, which is similar to Cronbach α value of the original scale [32]. Internal consistency maintained the same value even if any item was deleted. The assessment of reliability over time involved a retest conducted 2 weeks after the initial contact, and the ICC was used for calculation. The ICC values ranged from 0.504 to 1.000, with an overall value of 0.974. This value surpassed the reliability values obtained in 2 comparable studies conducted on the Chinese population (0.950 and 0.939) [18,51]. These results demonstrate that the European Portuguese version of the GFI exhibits excellent reliability over time, confirming its consistency as a measurement tool. The factor analysis yielded the same number of factors as the original scale, indicating that the translated version maintains a similar underlying factor structure to the original scale, suggesting that it measures the same fundamental constructs or dimensions as the items in the original version [52]. This encouraging finding indicates that the translation process effectively retained the core structure of the scale. Moreover, this confirmation of reliability and validity further demonstrates that the translated version of the GFI effectively preserves the essential elements of the original instrument.

The general hypothesis of this study was that a smartphone GFI app would show the same validity and reliability as the paper version, and it was proved in this study. The interinstrument reliability was studied to investigate the agreement and consistency between the 2 instruments, thereby ensuring the robustness of the results. High interinstrument reliability strengthens the credibility and accuracy of research outcomes, contributing to the overall quality and validity of assessments and evaluations. In this study, high levels of agreement were recorded between the instruments, proving that the digital format of the questionnaire is as reliable as the paper version. The reliability assessment over time was done in the same way as for the paper version of the GFI. The ICC values ranged from 0.589 to 0.958, with an overall value of 0.800, evidencing that, just like on paper, the digital version of the GFI remained reliable over time. The validity of the application was assessed by comparing the answers and total scores of the same individuals in the paper format (conventional) and the application (the new format to be validated). The results revealed a robust, positive, and statistically significant relationship between the responses in both forms. These findings not only establish the reliability of the application but also demonstrate its validation.

Health apps are vital in modern health care, providing easy access to health information, monitoring tools, and personalized advice [53]. The importance of mHealth becomes clear when examining its impact on health care accessibility. Many people, especially those living in disadvantaged or remote regions, face significant challenges in accessing health services. mHealth serves as a powerful equalizer, enabling individuals to connect with health care professionals, get immediate medical advice, and access health-related information, regardless of their geographic location [54]. This level of accessibility empowers patients and fosters early intervention and preventative care, ultimately resulting in improved health outcomes [55]. However, the proliferation of health apps on smartphone-based app stores is still on the rise due to the ease of app development, the absence of regulation, and the direct marketing to users, leading to an overcrowded scenario, which lacks reliability and accuracy. However, the ability of health stakeholders to discern the quality of these apps still lags significantly. This challenge largely stems from the lack of validation, which hinders app reliability and validity assurance [56]. Some potential benefits of validating health apps include improved accuracy and reliability of the app, increased user confidence in the app, and improved safety and effectiveness [57,58].

Assessing frailty is crucial as it helps identify individuals at higher risk of adverse health outcomes and enables timely interventions to mitigate these risks. Moreover, frailty assessments may guide treatment decisions, leading to tailored health care approaches focused on maintaining independence and managing chronic conditions. The impact of frailty in individuals and communities is profound, increasing health care usage, decreasing quality of life, and adding strain on families and caregivers. Understanding these effects allows for prioritized interventions, effective resource allocation, and appropriate support, ultimately improving the well-being and outcomes of frail individuals. The advantages of using apps for frailty assessment include their ease of use, ability to provide real-time feedback to the user, and their potential for improving the accuracy and reliability of frailty assessment [59,60]. An app designed to assess frailty offers numerous advantages and can revolutionize how frailty is identified. Its accessibility on smartphones and digital devices ensures easy and convenient frailty assessment, even in remote areas, while enabling early detection of an individual’s physical and cognitive abilities, allowing for timely interventions.

However, a key challenge in implementing mHealth solutions for frailty is the digital divide. Frail individuals, especially those from disadvantaged backgrounds, may lack access to necessary technology. This gap exacerbates health inequalities, as those in need of mHealth interventions the most may have the least access to them [61]. Ensuring fair access to technology and mHealth tools is a critical ethical issue [62]. Creating mHealth apps and devices that are easy to use and accessible to frail individuals is essential. This involves overcoming challenges related to age-related impairments in vision, hearing, and dexterity, which were considered in the development of the FRAILSURVEY [63].

The advancements in mHealth for frailty care hold the potential for deeply personalized interventions. Using cutting-edge data analytics and artificial intelligence, mHealth platforms will be adept at customizing interventions according to the distinct needs and characteristics of each frail individual [64]. The integration of mHealth platforms with electronic health records holds the potential to enhance care coordination and facilitate seamless data sharing. This interoperability will streamline processes, improve communication, and, ultimately, raise the standard of service. Furthermore, the evolving landscape of mHealth in frailty care will require ongoing ethical dialogues. As technological capabilities progress, ethical dilemmas around privacy, data security, informed consent, and fairness in algorithms will persist as paramount concerns. Navigating the delicate balance between innovation and ethical protection will present a constant challenge [65,66].

This study has some limitations that should be highlighted. Despite using a randomized, repeated crossover design and recruiting 522 participants from diverse settings, including community-dwelling older adults and nursing home residents, limitations persisted, only a fraction of participants underwent test-retest analysis, reducing the robustness of the results. In addition, the digital literacy barrier among older adults may have impacted the accessibility of the smartphone-based app, FRAILSURVEY. Furthermore, biases inherent in translating and validating scales into different languages may have influenced the study results. However, the similarity of psychometric properties to previous validation studies and the pioneering demonstration of the validity of the smartphone version offer promising insights into frailty assessment methods. This study marks a significant contribution to the field by establishing the scientific validity of the smartphone version of the GFI.

### Conclusions

Overall, FRAILSURVEY uses a standardized and validated assessment tool, providing objective and consistent measurements, eliminating subjective biases, and enhancing accuracy and reliability. Compared with traditional in-person assessments, its cost-effectiveness can reduce health care expenses and increase accessibility. The future integration with electronic health records and other health-related data could enable personalized interventions tailored to an individual’s specific needs and medical history, ultimately leading to more effective frailty management and improved overall health outcomes for at-risk individuals.

## Appendix

Multimedia Appendix 1 Final version of the European Portuguese version of the Groningen Frailty Indicator.

## Abbreviations

GFI: Groningen Frailty Indicator
ICC: intraclass correlation coefficient
mHealth: mobile health
PRISMA-7: Program on Research for Integrating Services for the Maintenance of Autonomy questionnaire

## Data Availability

The dataset generated during this study is not publicly accessible due to ethical considerations. However, both the dataset and the study metadata can be obtained from the corresponding author upon request.
