# Supplementary material for: FRAILSURVEY—an mHealth App for Self-Assessment of Frailty Based on the Portuguese Version of the Groningen Frailty Indicator: Validation and Reliability Study
Source: JMIR Form Res. 2025 Mar 7;9:e51975. doi: 10.2196/51975 (PMC11928775; doi:10.2196/51975)
Supplement: Multimedia Appendix 1 [file formative_v9i1e51975_app1.docx]

Table S1 Multimedia Appendix 1. Final version of the European Portuguese Version of the Groningen Frailty Indicator.

| Indicador de Fragilidade de Groningen—Versão Português Europeu |
| --- |
|  |
| É capaz de levar a cabo estas tarefas de forma independente? (A utilização de recursos como uma bengala, um andarilho, uma cadeira de rodas, é considerado independente) |
| 1. Compras |
| 1. Andar no exterior (à volta da casa ou nas redondezas) |
| 1. Vestir-se e despir-se |
| 1. Ir à casa de banho |
| 1. Que pontuação é que dá à sua própria capacidade física (0 a 10)? |
| 1. Tem dificuldades na vida diária devido à falta de visão? |
| 1. Tem dificuldades na vida diária devido a dificuldades auditivas? |
| 1. Durante os últimos 6 meses perdeu involuntariamente muito peso? (3kg num mês ou 6kg em 2 meses)? |
| 1. Toma 4 ou mais tipos diferentes de medicamentos? |
| 1. Tem alguma queixa relacionada com a sua memória? |
| 1. Sente, por vezes, um vazio à sua volta? |
| 1. Sente, por vezes falta de pessoas à sua volta? |
| 1. Sente-se abandonado? |
| 1. Sentiu-se, recentemente, abatido ou triste? |
| 1. Sentiu-se, recentemente, nervoso ou ansioso? |
| Scoring |
| Questões 1-4: Sim = 0; Não = 1 |
| Questão 5: 0-6 = 1; 7-10 = 0 |
| Questões 6-15: Não = 0; Sim = 1 |
